# Anti-cyclic citrullinated peptide antibodies in psoriatic arthritis – cross-sectional study and literature review

**Published:** 2013-12-25

**Authors:** C Popescu, S Zofotă, V Bojincă, R Ionescu

**Affiliations:** * "Sfânta Maria" Clinical Hospital, Bucharest; ** "Carol Davila" University of Medicine and Pharmacy, Bucharest

**Keywords:** anti-cyclic citrullinated peptide antibodies, anti-CCP antibodies, psoriatic arthritis

## Abstract

Abstract

Rationale: Anti-CCP antibodies are detectable not only in rheumatoid arthritis (RA), but also in psoriatic arthritis (PsA). It is possible those anti-CCP antibodies are associated with features of PsA and that these auto-antibodies are useful in distinguishing PsA from RA.

Objective: to evaluate the prevalence and the associations of anti-CCP antibodies in PsA patients; to evaluate the usefulness of anti-CCP antibodies in distinguishing PsA from RA.

Methods and Results: The inquiry was designed as a cross-sectional study of 41 PsA patients, 139 RA patients and 147 normal subjects, which recorded demographic data, disease activity and serology: rheumatoid factor (RF), anti-CCP antibodies. Five PsA patients (12.2%) were anti-CCP positive. Compared to anti-CCP negative PsA patients, anti-CCP positive PsA patients had a more frequently a polyarticular disease pattern (p = 0.005), they were more frequently treated with biologics (p = 0.015) and less frequently with classic disease-modifying drugs (p < 0.001). An optimal positive cutoff value for anti-CCP titer was determined (11.6 U/mL), over which it is highly probable that a known PsA patient actually has RA and psoriasis.

Discussion: The more aggressive the disease of anti-CCP positive PsA patients indicates the need of a more intensive management regarding anti-rheumatic treatment and follow-up. Anti-CCP antibodies can be a useful tool in differentiating PsA from RA, especially in RA-like forms of PsA, which present no elements pertaining to spondyloarthropathies.

Abbreviations: anti-CCP - anti-cyclic citrullinated peptide antibodies; ACR - America College of Rheumatology; CRP - C-reactive protein; CASPAR - The Classification Criteria for Psoriatic Arthritis; DMARD – disease modifying anti-rheumatic drug; EULAR - European League against Rheumatism; ELISA - enzyme-linked immunosorbent assay; ESR - erythrocyte sedimentation rate; HLA – human leukocyte antigen; PsA - psoriatic arthritis; RA - rheumatoid arthritis; RF - rheumatoid factor; ROC - receiver operating characteristic.

## Introduction

Psoriatic arthritis (PsA) is a chronic inflammatory disease in which arthritis is associated in most cases with psoriasis. The biological and clinical spectrum of PsA may present common elements with rheumatoid arthritis (RA; e.g. symmetrical arthritis of the hands, elevated acute phase proteins) or with the general class of spondylarthropathies (e.g. dactylitis, enthesitis, sacroiliitis). Unfortunately, there is no specific serologic test for PsA. Rheumatoid factor (RF) contributed to the designation of PsA as an independent nosological entity, in the sense that patients with arthritis and psoriasis were usually seronegative for RF, differentiating them from RA patients, who are usually seropositive for RF, but its low specificity for RA motivated the search for a more reliable serologic test. Anti-cyclic citrullinated peptide antibodies (anti-CCP) met the demands: they proved a similar sensibility for RA (55-80%), but a higher specificity (96-98%) [**1**]. As a consequence, anti-CCP antibodies were included in the RA classification criteria [**2**]. Anti-CCP antibodies are mainly produced in the synovium by the local plasma cells [**3**], and are designed to bind to citrulline-containing antigenic determinants of synovial proteins. The enzyme peptidyl-arginine-deiminase generates citrulline residues by acting on the normal arginine residues [**4,5**]. In clinical practice, the titer of anti-CCP antibodies is determined by an enzyme-linked immunosorbent assay (ELISA), using synthetic citrullinated peptides. The detection of anti-CCP antibodies may precede by several years the clinical onset of RA [**6**], for which they have a high positive predictive value and a strong association with female gender [**7**], disease activity [**8**], functional impairment and erosive disease [**9,10**]. The studies which evaluated anti-CCP antibodies in PsA patients reported a prevalence of 5.6-20% [**7,11-17**]. In this context, the present study aims to evaluate the prevalence of anti-CCP antibodies in a PsA group, their clinical usefulness and their possible correlations with clinical and laboratory parameters.

## Discussion

Patients

 Following a cross-sectional prospective design, the randomly selected population sample was divided in three groups according to the diagnosis. The test group comprised 41 PsA in-patients, who met the CASPAR 2008 classification criteria of PsA [**18**]. The positive control group comprised 139 RA in-patients, who met the ACR/EULAR 1987 and/or 2010 classification criteria of RA [**2,19**]. The negative control group comprised 147 normal subjects, who did not present any auto-immune or chronic inflammatory disease. All the participants in the study gave informed consent to blood sample collection and data usage in medical research purposes. The study was approved by the local ethics committee.

 Laboratory measurements

Five milliliters of blood were obtained by venipuncture at the time of routine blood tests collection. The titer of anti-CCP antibodies was measured by an ELISA technique, using a commercial available kit (Anti-CCP, Abbott Architect System, Dundee, United Kingdom), according to the manufacturers’ recommendations which stated a positive cutoff value of 5 U/mL. Erythrocyte sedimentation rate (ESR) was determined by the Westergren technique, with a positive cutoff value at 30 mm/h. RF titer and C-reactive protein (CRP) concentration were determined by immunonephelometry, with normal values under 30 U/ml and 5 mg/L respectively.

 Statistical analysis

 Normally distributed data were reported as means with standard deviation and range, while non-normally distributed data were reported as medians and range. Qualitative data were reported as percentages and absolute values. The differences between groups were evaluated using non-parametric tests: χ2 for nominal variables and Mann-Whitney U test for scale variables. Correlation was established by computing Spearman coefficients. Sensitivity and specificity of anti-CCP antibodies were studied using receiver operating characteristic (ROC) curves. All the statistical tests were 2-sided, were considered significant at p ≤ 0.05 and were computed with SPSS Statistics v.17.0.1 for Windows (SPSS Inc., Chicago, S.U.A., 2008).

## Results

Group characteristics

Table 1 summarizes the general characteristics of the studied groups. The patients with PsA and the normal subjects presented an average titer of anti-CCP antibodies below the positive cutoff of the test (a median of 2 U/mL in the PsA group and a mean of 2.5 U/mL in the normal group). This observation is explained by the fact that the titer of individuals positive for anti-CCP antibodies was relatively small compared to the positive cutoff value (e.g. 9.2 U/mL). The patients with RA presented an average titer of anti-CCP antibodies well beyond the positive cutoff (a median of 14 U/mL), which can be explained by the fact that some of these patients had very high titers compared to the positive cutoff (e.g. 200 U/mL).

**Table 1:** Group characteristics.*

diagnosis	psoriatic arthritis	rheumatoid arthritis	normal subjects
count	41	139	147
female	23 (56.1%)	122 (87.8%)	124 (84.4%)
male	18 (43.9%)	17 (12.2%)	23 (15.6%)
age [years]	50.2 ± 14.2	58.1 ± 13.4	54.4 ± 13.9
(range)	(23 - 77)	(19 - 81)	(20 - 89)
disease duration [months]	48	72	-
(range)	(1 - 288)	(0,5 - 480)	
ESR [mm/h]	39.3 ± 26.9	38.7 ± 26.2	29.9 ± 22.9
(range)	(2 - 118)	(2 - 109)	(2 - 102)
CRP [mg/L]	10	8.4	4.8
(range)	(0.14 - 84)	(0.2 - 197)	(0.2 - 320)
RF positive	2 (4.9%)	92 (66.2%)	7 (4.7%)
RF titer [UI/mL]	10.9 ± 7,8	56	14.3 ± 10.8
(range)	(2.2 - 42)	(4.8 - 953)	(3.4 - 106)
anti-CCP positive	5 (12.2%)	84 (60.4%)	2 (1.4%)
anti-CCP titer [UI/mL]	2	14	2.5 ± 1.5
(range)	(0.1 - 27)	(0.1 - 200)	(0.1 – 5.8)
polyarticular involvement&	17 (41.5%)	139 (100%)	0
glucocorticoids treatment	3 (7.3%)	44 (31.6%)	0
DMARD treatment	35 (85.4%)	122 (87.8%)	0
biologic treatment	4 (9.8%)	6 (4.3%)	0
Notes: * numeric data are reported either as means with standard deviation and interval, or as medians with extremes. & polyarticular involvement = 5 or more affected joints.

Anti-CCP antibodies

**Fig. 1A:**
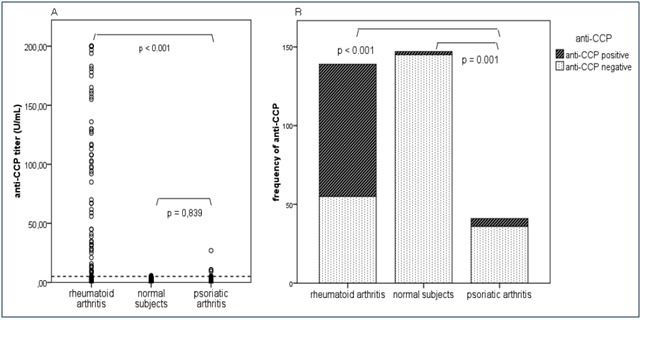
The distribution of anti-CCP titers according to the diagnosis. The dotted line represents the positive cutoff value (5 U/mL). B. The distribution of anti-CCP positivity and negativity according to the diagnosis

The frequencies of anti-CCP positive patients were distributed as follows: 5 PsA patients (of 41; 12.2%), 84 RA patients (of 139; 60.4%) and 2 normal subjects (of 147; 1.4%). It is possible that the total number of positive anti-CCP subjects is underestimated, since 128 RA patients (92.1%) and 35 PsA patients (85.4%) were receiving classical disease-modifying antirheumatic drugs (DMARD), which were proved to lower the anti-CCP antibodies titer [**8**]. The differences between positive and negative anti-CCP frequencies among the three groups were statistically significant (p < 0,001; Table 2). In the same manner, statistically significant differences were observed between the mean titers of anti-CCP antibodies of the three groups. Analyzing the groups two by two (**Fig. 1**), we observed that the titers of anti-CCP antibodies of PsA patients (rank = 48.3) were significantly different from the titers of anti-CCP antibodies of RA patients (rank = 102.9; U = 1118.5; p < 0,001), in accordance with the frequency of positive anti-CCP titers which also were significantly different (χ2(1, n = 180) = 29.47; p < 0,001). Instead, compared to the anti-CCP titers of normal subjects (rank = 94.9), the titers of anti-CCP antibodies of PsA patients (rank = 92.9) were not significantly different (p = 0.839), in spite of the fact that the frequency of positive anti-CCP titers did differ significantly (χ2(1, n = 188) = 10.5; p = 0.001).

**Table 2:** Differences between frequencies and titers of positive and negative anti-CCP antibodies among the three groups.

anti-CCP	psoriatic arthritis (n = 41)	rheumatoid arthritis (n = 139)	normal subjects (n = 147)	p*
frequency				
positive (n)	5	84	2	
negative (n)	36	55	145	< 0.001&
mean titer	positive (U/mL; range)	12.6 (5.5 - 27)	122.4 (5.4 - 200)	5.6 (5.4; 5.8)
negative (U/mL; range)	2.1 (0.1 - 4.9)	2.7 (0.1 - 4.8)	2.4 (0.1 - 4.9)	0.004#
Notes: * calculated with the χ2 test (significant if p ≤ 0,05); & χ2(2, n = 327) = 129.83; p < 0.001; # χ2(2, n = 148) = 11.27; p = 0.004.

**Fig. 2:**
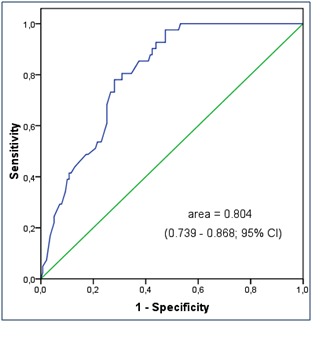
ROC curve of titers of anti-CCP in patients with RA and PsA

The differences obtained between anti-CCP titers in patients with RA and PsA indicated the evaluation of the value this test has in discriminating between the two illnesses, supposing a pre-test probability of 50%. The area under the ROC curve was 0.804 (0.739 – 0.868; 95% confidence interval; standard error of 0.033 under non-parametric assumption), which advocates the fact that anti-CCP antibodies are a good test for this purpose (p < 0.001). The optimal cutoff positive titer, under which a PsA diagnosis can be sustained, was determined to be 11.6 U/mL (97.6% sensitivity, 52.5% specificity; 95% CI – Fig. 2).

There were no significant correlations between the concentration of anti-CCP antibodies and the reviewed clinical and laboratory parameters in the PsA and normal groups. Instead, in the RA group, the titer of anti-CCP antibodies correlated significantly with the titer of RF (r = 0.497; p < 0.001) and with the ESR (r = 0,182; p = 0,032), suggesting that they arise from the same pathological inflammatory response which accounts for auto-immunity and for the production of acute phase proteins. Some studies report a slight correlation between anti-CCP and RF concentrations or RA patients [**14,20**], but others do not find this correlation [**8,21,22**].

Concerning the demographic and clinical data of PsA patients (Tables 3 and 4), the anti-CCP positive and anti-CCP negative subgroups were significantly different in terms of anti-rheumatic treatment and of joint involvement patter. Patients with PsA and positive anti-CCP antibodies had a lower frequency of classical DMARD treatment when compared with anti-CCP negative PsA patients (60% and 88.9% respectively; p < 0,001) and a higher frequency of treatment with biologic anti-inflammatory drugs (40% and 5.6% respectively; p = 0,015). All of the 5 anti-CCP positive patients had a polyarticular disease pattern (five or more affected joints), compared to only 12 anti-CCP negative patients (33%; p = 0,005). 

**Table 3:** Subgroup analysis of PsA patients according to anti-CCP positivity

	anti-CCP⊕ (n = 5)	anti-CCP⊖ (n = 36)	p*
male n (%)	1 (20%)	17 (47.2%)	0.250
female n (%)	4 (80%)	19 (52.8%)	
age ± SD (years)	48.2 ± 7.2	50.5 ± 14.9	0.750
(range)	(39-55)	(23-77)	
disease duration ± SD (months)	36	54	0.749
(range)	(4-288)	(1-256)	
ESR ± SD (mm/1h)	30 ± 7.1	40.6 ± 28.5	0.437
(range)	(20-36)	(2-118)	
CRP ± SD (mg/L)	4	10.65	0.157
(range)	(0.1-20)	(1.2-84)	
RF ± SD (U/mL)	10.6	10.3 ± 6.1	0.704
(range)	(2.6-42)	(2.2-38)	
anti-CCP ± SD (U/mL)	12.6 ± 8.3	2.1 ± 1.4	< 0.001#
(range)	(5.5-27)	(0.1-4.9)	
polyarticular disease n (%)	5 (100%)	12 (33.3%)	0.005$
DMARD treatment n (%)	3 (60%)	32 (88.9%)	< 0.001&
glucocorticods treatment n (%)	1 (20%)	2 (5.6%)	0.245
biologic treatment n (%)	2 (40%)	2 (5.6%)	0.015§
Notes: - when appropriate, numeric values were reported either as means with standard deviation (SD) or as medians with range. * the differences between sex, treatment and involved joint pattern were evaluated with the χ2 test; the differences between the other variables were evaluated with the Mann-Whitney U test, both being significant at p ≤ 0,05; # Z = - 3.59, p < 0.001; $ χ2(1, n = 41) = 8.04; p = 0.005; & χ2(3, n = 41) = 41; p < 0.001; § χ2(1, n = 41) = 5.92; p = 0.015

**Table 4:** Characteristics of normal and PsA patients positive for anti-CCP antibodies

diagnosis	psoriatic arthritis						normal
patient	A	B	C	D	E	F	G
sex	F	F	F	M	F	F	F
age (years)	53	39	52	55	42	70	57
disease duration (months)	288	6	36	144	4	-	-
ESR (mm/1h)	36	35	25	34	20	35	45
CRP (mg/L)	20	11	1.6	4	0.14	6.4	6.8
RF (U/mL)	42	10.6	2.6	17	5.9	6	42
anti-CCP (Um/L)	27	9.2	5.5	10.9	10.3	5.4	5.8
polyarticular disease	yes	yes	yes	yes	yes	-	-
treatment	δ + γ	δ	β	β	δ	-	-
Abbreviations: F – female; M – male; δ – disease modifying anti-rheumatic drug; γ – glucocorticoids; β –biologic anti-inflammatory drugs.

## Discussion

The study recorded a 12.2% (5 of 41) prevalence of anti-CCP positivity among PsA patients. This prevalence is within the limits of the reported prevalence in the literature.

There were no differences between anti-CCP positive and anti-CCP negative PsA patients in terms of sex, age, disease duration, acute phase reactants (ESR, CRP), RF and treatment with glucocorticoids. On one hand, after testing the degree of association of the frequencies of positive and negative anti-CCP antibodies with RF titers in PsA patients reported in literature, we noted that the majority of studies recorded a significant association between these serologic markers (p ≤ 0,05) [**7,13,14,16,23**]. The lack of this association in our study is in agreement with the results of Korendowych, Candia and Fattah [**12,15,24**]. On the other hand, compared to the anti-CCP negative PsA patients, anti-CCP positive PsA patients had a higher number of involved joints, a lower frequency of DMARD treatment and a higher frequency of treatment with biologics. The association of positive anti-CCP antibodies with the polyarticular disease pattern confirms the results of Candia, Fattah and Maejima [**15,17,24**], and disaffirms the results of Inanc and Korendowych [**12,14**], who did not report a significant association, but found that positive titers of anti-CCP antibodies can be detected in PsA patients with oligoarticular, asymmetric disease pattern, phenotipically different from RA. This contradiction may have its origin in the small sample size analyzed by some of the cited authors. The prevalence of the polyarticular disease pattern in anti-CCP positive PsA patients varies among the different studies. Our 100% prevalence is in agreement with the results of Inanc et al. [**14**], Maejima et al. [**17**], and Fattah et al. [**24**]. Other authors also found high prevalence rates, for example 86.7% in the Vander-Cruyssen study [**13**], 72.7% in the Alenius study [**7**], 72,4% in the Candia study [**15**]. Lower prevalence was also reported: 57.1% in the Korendowych study [**12**], 56.3% in the Bogliolo study [**11**], 50% in the Ouedraogo and Shibata studies [**16,23**]. The data regarding the associations of anti-CCP antibodies with the type of treatment for PsA are again contradictory: for example Korendowych, Bogliolo şi Maejima support this association with classical DMARDs [**11,12,17**], while Inanc and Vander-Cruyssen disaffirm it [**13,14**]. Vander-Cruyssen et al. found no association of ant-CCP antibodies with biological [**13**]. In our study, the association of anti-CCP antibodies with glucocorticoid treatment was not significant, in contrast with the Inanc study [**14**]. There are multiple sources of these discrepancies, including differences between clinicians’ therapeutic strategies, variable compliance to treatment and patient access to medical assistance. A prospective study, using a bigger sample and a longer follow-up period would be more likely to clarify the association between anti-CCP antibodies, type of treatment and disease pattern of PsA patients.

Among the PsA patients with positive anti-CCP antibodies (**Table 4**), patient A also presented a positive titer of RF. It is possible that this case carries a false PsA diagnosis, since it has two key features suggestive for RA: positive serology (anti-CCP, RF) and polyarticular symmetric joint involvement. A coincidence of RA and psoriasis would then be a more appropriate diagnosis. Although the classification criteria of PsA require the absence of RF, we considered that its presence cannot differentiate in this case between PsA and RA with psoriasis, since RF is detectable in 5% of normal individuals [**19**], even more in the aging population [**25**], in non-PsA patients with different chronic and infectious diseases, but also and in 5-10% of PsA patients [**26,27**]. One can diagnose with PsA a RF-positive patient, especially if he or she has specific disease traits (e.g. dactylitis). Patient A did not present any of these suggestive clinical features; therefore we opted for a RA diagnosis. An extra argument in favor of this option is that the anti-CCP titer of this patient was at least two times higher than optimal cutoff value calculated in our group (11.6 U/mL). The high sensitivity (97.6%) of an anti-CCP titer lower than 11.6 U/mL to sustain the diagnosis of a PsA patient allows us to assert that it is highly probable that a patient diagnosed with PsA, whose titer of anti-CCP antibodies surpasses the optimal cutoff, actually has RA and psoriasis. The doubt remains in the case of PsA patients whose anti-CCP titer is between 5 and 11.6 U/mL, in which interval one cannot exclude RA because of the low specificity of the anti-CCP test (52.5%).

Patients with PsA differed from normal subjects by the frequency of positive anti-CCP titer, not by the titers themselves. The reason for this is that the positive anti-CCP titers of PsA patients in our study were generally situated around two times the normal value, which is much smaller in comparison to titers recorded for RA patients and even in comparison to the ones reported in other groups of PsA patients [**16**]. In this context, we could have gained more information regarding the background of the presence of anti-CCP antibodies in PsA patients if we could examine the genetic terrain of these anti-CCP positive patients, knowing that Korendowych et al. observed that all PsA patients with positive anti-CCP antibodies carried the common epitope (HLA-DRB1) [**12**], a genotype characteristic to RA [**21**]. The data regarding the genetic terrain of PsA patients were not available for this study.

Not least, one should note that positive anti-CCP antibodies may occur in normal subjects, less frequently than in RA patients. One of control subjects, included in the normal group (patient G, Table 4), was being monitored for sensitive polineuropathy, unilateral sacroiliitis (HLA B27 absent), positive titers of RF, antinuclear, anti-U1 RNP and anti-CCP antibodies, with no signs and symptoms of arthritis, both at the time of the study and in her medical history. It its possible that this patient was evaluated serologically in the window between the appearance of auto-antibodies and the clinical onset of RA. Since the design of the study was cross-sectional, anti-CCP positive patients were not monitored to record the polyarticular disease pattern in PsA patients or the onset of RA in normal subjects. Another drawback of the study is that it did not evaluate the association between anti-CCP antibodies and radiological elements of active PsA (e.g. erosions), knowing that previous studies noted the significant association of positive titers of anti-CCP antibodies with the presence of erosive PsA [**11**].

**Conclusions**

The frequency of anti-CCP positivity in PsA patients was much lower compared to RA patients and higher compared to normal subjects, while the actual titer of anti-CCP antibodies differed significantly only in comparison with RA patients, being equivalent in rank with the anti-CCP titers of normal subjects. Judged in clinical context, a known PsA patient, who scores a titer of anti-CCP antibodies higher that 11.6 U/mL, will most likely have RA and psoriasis at the same time. For this reason, it may be useful to quantify the concentration of anti-CCP antibodies for all known PsA patients.

The anti-CCP titers of PsA patients were not correlated with any of the clinical and laboratory variables recorded. Compared to anti-CCP negative PsA patients, anti-CCP positive PsA patients presented more frequently a polyarticular disease patter and received more frequently biological anti-inflammatory drugs and less frequently classical DMARDs. The fact that the anti-CCP positive subgroup of PsA patients presented a more aggressive disease indicates the need of a more intense management with regard to disease-modifying treatment and follow-up.

**Acknowledgements**

The authors gratefully thank Associate Professor Dr. Cristian Băicuş for his excellent statistical remarks.

**Conflicts of interest**

None declared.
